# Seasonal Variability in Calorimetric Energy Content of Two Caribbean Mesophotic Corals

**DOI:** 10.1371/journal.pone.0151953

**Published:** 2016-04-06

**Authors:** Viktor W. Brandtneris, Marilyn E. Brandt, Peter W. Glynn, Joanna Gyory, Tyler B. Smith

**Affiliations:** 1 Center for Marine and Environmental Studies, University of the Virgin Islands, St. Thomas, United States Virgin Islands; 2 Rosenstiel School for Marine and Atmospheric Sciences, University of Miami, Miami, Florida, United States of America; 3 Department of Ecology and Evolutionary Biology, Tulane University, New Orleans, Louisiana, United States of America; Biodiversity Research Center, Academia Sinica, TAIWAN

## Abstract

Energetic responses of zooxanthellate reef corals along depth gradients have relevance to the refugia potential of mesophotic coral ecosystems (MCEs). Previous observations suggested that MCEs in the Caribbean are thermally buffered during the warmest parts of the year and occur within or just below the chlorophyll maximum, suggesting abundant trophic resources. However, it is not known if mesophotic corals can maintain constant energy needs throughout the year with changing environmental and biological conditions. The energetic content of tissues from the stony coral species *Orbicella faveolata* and *Agaricia lamarcki* was measured on the southern insular shelf of St. Thomas, US Virgin Islands (USVI), using micro-bomb calorimetry. Three sites for each species, at depths of 6m, 25m, 38m and 63m, were selected to capture energetic differences across the major vertical range extent of both species in the USVI—and sampled over five periods from April 2013 to April 2014. Mesophotic colonies of *O*. *faveolata* exhibited a significant reduction in energetic content during the month of September 2013 compared to mid-depth and shallow colonies (p = 0.032), whereas *A*. *lamarcki* experienced similar energetic variability, but with a significant reduction in energy content that occurred in July 2013 for colonies at sites deeper than 25m (p = 0.014). The results of calorimetric analyses indicate that *O*. *faveolata* may be at risk during late summer stress events, possibly due to the timing of reproductive activities. The low-point of *A*. *lamarcki* energy content, which may also coincide with reproduction, occurs prior to seasonal stress events, indicating contrasting, species-specific responses to environmental variability on MCEs.

## Introduction

Dramatic changes in the physical parameters of the ocean are predicted to increase mortality of corals and organisms associated with coral reefs [1–3]. Many of the studies undertaken to elucidate the effects of myriad stressors—including increased temperatures, ocean acidification and anthropogenic disturbance—on corals have suggested that coral reefs are at great risk of loss and possible extinction in the future [4–6]. Recently, a somewhat more positive outlook on the future of coral reefs has been promoted by those investigating the refuge potential of deep, light-dependent coral habitats—referred to as mesophotic coral ecosystems (MCEs) [7–9]. MCEs are defined as reef ecosystems comprised of phototrophic and azooxanthellate scleractinian corals, sponges and macroalgae between 30m and the depth at which light in the water column is too low to sustain photoautotrophy, perhaps as deep as 150m depending on local light attenuation [10–12]. Depth generalist coral species inhabiting both shallow and mesophotic reefs may experience widely variable conditions dependent on location and season [12–13]. Often located offshore, MCEs may experience unique thermal, light, salinity and sedimentation regimes compared to their shallow, nearshore counterparts [11–12]. Deeper water may provide a protective buffer for corals against increased temperature, storm-induced wave action and UV radiation [14–16]. The “deep reef refugia” hypothesis suggests that MCEs sheltered from increased temperature and wave action have the potential to support healthy coral that can provide larvae for the repopulation of degraded shallow water coral ecosystems [7,17].

The potential for MCEs to serve as coral refugia in the face of climate change depends largely on the ability of corals beyond 30m to persist through increasingly prevalent stress events. It has been shown that coral colony energy content can play an important role in the ability of corals to survive and recover from intense stress events [18–24]. Energy content—in this case lipid content—has been used to accurately predict survivorship of laboratory colonies exposed to a range of temperature, light and sedimentation [20]. Not only does energy content at time of bleaching greatly influence the survivorship of corals, the ability of colonies to increase heterotrophic feeding post-bleaching has been shown to increase resilience in at least one coral species subjected to thermally induced bleaching [19, 24]. Corals that are able to supplement reduced autotrophic energy production by suspension feeding on particulate matter may be more likely to survive prolonged bleaching events [21–24]. Heterotrophic plasticity, however, is based on both the coral species in question and the presence of coral food sources in the water column [13, 25–27].

Several different techniques can be used to measure the physiological and energetic status of corals. Along with lipid content and isotopic analyses, measures of tissue biomass and zooxanthellae type and density are widely used to inform the energetic quality and stress susceptibility of corals [18,20,26,28–32]. Another technique not often utilized in modern reef study is coral calorimetry. First applied to corals by Richmond, calorimetry is a direct measure of the total energy within a coral holobiont [33]. The reductive nature of this methodology provides a single measure of energy content that can be easily compared through space and time as well as across species. The technique is limited, however, in that the energy content measured is that of the overall pool of energy in a colony, and does not provide information on the sources of incoming energy (i.e., heterotrophy versus autotrophy) or causes of energy loss. The energy available for growth, reproduction and physiological maintenance is contained within the overall measure of energetic content.

This study assesses seasonal changes in adult coral energetic status and measures seasonal energy changes across the depth ranges of two threatened Caribbean scleractinian corals, *Orbicella faveolata* and *Agaricia lamarcki*. We asked if energy content varied between shallow and mesophotic zones and whether this might influence the potential tolerance of these coral species to stress events. Five coral collections were made between April 2013 and April 2014 across three depths representing the primary habitat range for each species– 6 to 38m for *O*. *faveolata* and 25 to 63m for *A*. *lamarcki*. Calorimetric values at each depth were compared to seasonal measures of environmental characteristics to describe the relationship between depth, light, chlorophyll-a fluorescence—as a proxy for heterotrophic potential—and the energy content of both species.

## Materials and Methods

### Site Selection

**Fig 1:**
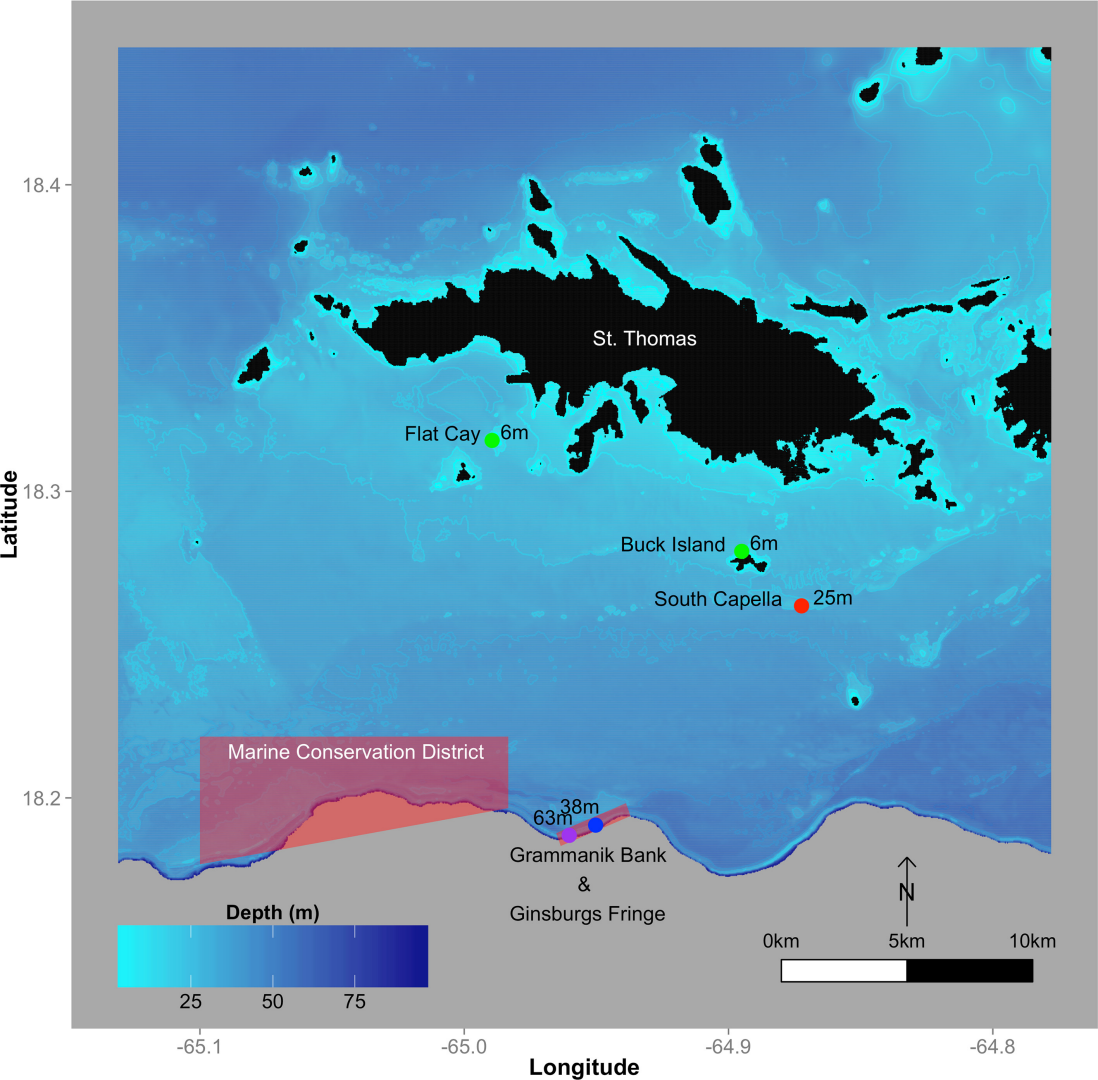
Study Area. Sampling locations on insular shelf south of St. Thomas, USVI. Major offshore Marine Protected Areas indicated in red shading. Land masses are colored in black. Green dots are used to indicate 6m sites, red for 25m, blue for 38m and purple for 63m.

All field work was conducted under permit ##DFW14017T issued by the Virgin Islands Department of Planning and Natural Resources. Sampling locations were chosen to encompass the primary depth range for each species (Fig 1; see [34] for GPS). Colonies of *O*. *faveolata* were sampled at sites in approximately 6, 25, and 38m of depth, while *A*. *lamarcki* was sampled at 25, 38, and 63m depth (Table 1). Initial shallow samples of *O*. *faveolata* taken on May 1, 2013 were from an offshore site at Buck Island where colony density was found to be very low. Therefore, subsequent shallow *O*. *faveolata* sampling was conducted at another offshore island, Flat Cay—deemed analogous to Buck Island due to similar environmental histories and distances from shore [data in S1 Supporting Information]. All sites sampled are included in the annual Territorial Coral Reef Monitoring Program (TCRMP) for the US Virgin Islands, providing consistent historic datasets for temperature and coral health [34].

**Table 1:** Site Information.

Site	Depth (m)	Species	Dates (N)
Buck Island	4–7	*O*. *faveolata*	1-May-13 (5)
Flat Cay	4–10	*O*. *faveolata*	11-Jul-13 (4), 13-Sep-13 (5), 19-Nov-13 (6), 2-Apr-14 (6)
South Capella	23–28	*O*. *faveolata/A*. *lamarcki*	1-May-13 (5/5), 11-Jul-13 (4/5), 13-Sep-13 (5/4), 19-Nov-13 (5/6), 2-Apr-14 (6/7)
Grammanik Bank	36–40	*O*. *faveolata/A*. *lamarcki*	26-Apr-13 (4/5). 5-Jul-13 (4/4), 18-Sep-13 (5/6), 14-Nov-13 (5/5), 4-Apr-14 (5/6)
Ginsburgs Fringe	60–67	*A*. *lamarcki*	26-Apr-13 (5), 5-Jul-13 (5), 18-Sep-13 (6), 14-Nov-13 (5), 4-Apr-14 (6)

Sampling sites with depth, species sampled, sampling dates and sample sizes.

### Coral Collection

Coral samples were collected over five periods between April 2013 and April 2014 at approximately two-month intervals (Table 1). Divers haphazardly sampled seven 15-30cm^2^ replicate, independent sections of each species separated by five fin-kicks while maintaining consistent depth at each site—producing a total of 105 samples for *O*. *faveolata* and 101 for *A*. *lamarcki* (Table 1). Colonies were not resampled during multiple collection periods. Hammer and chisel were used to collect from the tops of *O*. *faveolata* colonies and the colony edges of *A*. *lamarcki* with a minimum radius of 25cm. Colonies of *A*. *lamarcki* were not sampled from the center as this caused fracturing of the entire colony; however attempts were made to include as much of the central portion of the colony as possible. Lastly, divers recorded the collection depths, and length, width and height of each sampled colony. At the surface, samples were transferred without seawater to pre-labeled whirl-packs and placed on ice for transport back to the laboratory. Time constraints related to post-processing limited sampling to two sites per field day—concurrent samplings were carried out no more than seven days apart.

### Calorimetry

Coral samples were denuded with an airbrush according to the methods of Szmant and Gassman using ultra-pure 18mOHM water [35]. The blastate was homogenized and immediately frozen and stored at -20°C. Later, samples were partially thawed and transferred to lyophilization tubes before being re-frozen at -80°C for two hours. Samples were then freeze-dried for 24-36hrs at 220mbar and -105°C. Drying times were dependent on sample size and density—larger samples required longer drying times and in some cases re-freezing and a second round of lyophilization. Drying was deemed complete when samples could be easily powdered using a scapula without the presence of ice or liquid water. Powdered coral samples were stored in centrifuge tubes in a dehumidified cabinet set to 10% humidity.

Calorimetric analyses were carried out using a semi-microbomb calorimeter (Model 6725, Parr Instrument Company, Illinois, USA). Powdered coral samples weighing 8-24mg were pelletized and combined with a purified mineral oil spike of known energy density for combustion. Due to variable humidity in the laboratory it was difficult to consistently re-hydrate samples. The mineral oil spike ensured complete combustion of the coral powder and slowed the burn to an acceptable rate. Samples were loaded into the prepared microbomb and pressurized to 30atm with medical grade pure oxygen. Calorimetric analysis requires fifteen minutes per run and each sample was analyzed at least twice. Traditionally, relative standard deviation (RSD) between two or more calorimetry runs is used to ensure the accuracy of the final energetic content [36]. If the first two runs did not achieve an acceptable RSD, the sample was rerun until either an acceptable RSD was achieved or the sample was depleted. A minimum of 25mg freeze dried tissue was required for successful calorimetric analyses—74 samples of *O*. *faveolata* and 80 samples of *A*. *lamarcki* were sufficiently sized for calorimetric sampling.

Carbonate rich organisms present a unique problem in calorimetry due to the reduced combustion of calcium carbonate. Samples with >20% carbonate require a correction of 0.586 J/g carbonate [36–37]. 6-38mg of each sample was burned for 4 hours at 500C to ascertain carbonate percentage. In all cases, carbonate proportions were greater than 20% and required correction.

### Environmental Characterization

Continuous *in situ* records of temperature were recorded with sensors affixed to the substrate (Hobo Water Temperature Pro v2 U22, Onset Computer Corporation, Massachusetts, USA). Paired instruments at each site and at the coral sampling depths provided continuous temperature records at fifteen-minute intervals over the course of the study. Temperature probes were calibration checked pre- and post-deployment in a freshwater ice bath and ambient temperature bath, and probes were not deployed if their temperature deviated more than 0.3°C from that recorded with a bulb thermometer.

Vertical profiles of water column temperature, PAR and chl-a fluorescence were sampled within one month of coral collection dates using a Seabird 25 Conductivity-Temperature-Depth multi-sensor (Seabird Scientific, Washington, USA) equipped with an ECO-AFL/FL fluorometer (Wetlabs, Oregon, USA) sampling at a frequency of 8 Hz. Water column cross sections were taken at each sampling site as part of ongoing monitoring efforts. Additionally, *in situ* benthic chl-a fluorescence was sampled for one minute, every hour using an ECO-FLSB fluorometer (Wetlabs, Oregon, USA) at each of the mesophotic sites from September 21, 2013 to November 19, 2013.

### Analysis

Site specific measures of PAR, chl-a fluorescence and temperature were created for each sampling event by averaging CTD measurements within one meter of the coral sampling depth at each site. At South Capella the CTD sampling depths did not always reach the coral sampling depth at approx. 25m (3 of 5 measurements). In order to increase the sample size of physical variables we used a CTD sampling depth of 19m. Available data for two casts that retrieved data to 23m showed that the difference in physical variables between the depths for the same cast was small and well within the differences between casts at different sites. (mean differences in 23m to 19m depths July and November 2013, ΔPAR = -28.78 μmol s^-1^ m^-2^, Δchl-a = 0.11mg m^-3^, Δtemperature = -0.028°C). Multidimensional results were first visualized using a principal component analysis (PCA) followed by ANOVA comparison of each environmental characteristic to site. Additionally, seasonal water column stratification was investigated using CTD casts from the 63m Ginsburgs Fringe site.

Plotted benthic temperature records were condensed to daily means for each site and compared using repeated measures ANOVA (rm-ANOVA). In addition, the potential thermal stress experienced for a given site was calculated as the Degree Heating Week metric (DHW) [38]. Site-specific DHW calculations were based on derived bleaching thresholds for Flat Cay, South Capella, and Grammanik Bank [39]. No specific bleaching threshold is available for the deepest site, Ginsburgs Fringe (63m). A hypothetical bleaching threshold of 28.4°C was developed based on a relationship of bleaching threshold with depth from 24 sites of the Territorial Coral Reef Monitoring Program (Bleaching Threshold = 30.03°C—0.025°C * Depth in meters).

The change in energy content over the sampling periods was tested separately for *O*. *faveolata* and *A*. *lamarcki*. The independent nature of individual coral samples through time allowed for the application of a two-way ANOVA. Sampling Period and Site were used as factors and Tukey’s HSD post-hoc analysis was used to compare means when significant effects of the main factors were found. Regression analyses indicated no significant relationship between colony surface area and energy status for either species. Therefore, colony size was not considered in further statistical analyses.

## Results

### Calorimetry

**Fig 2:**
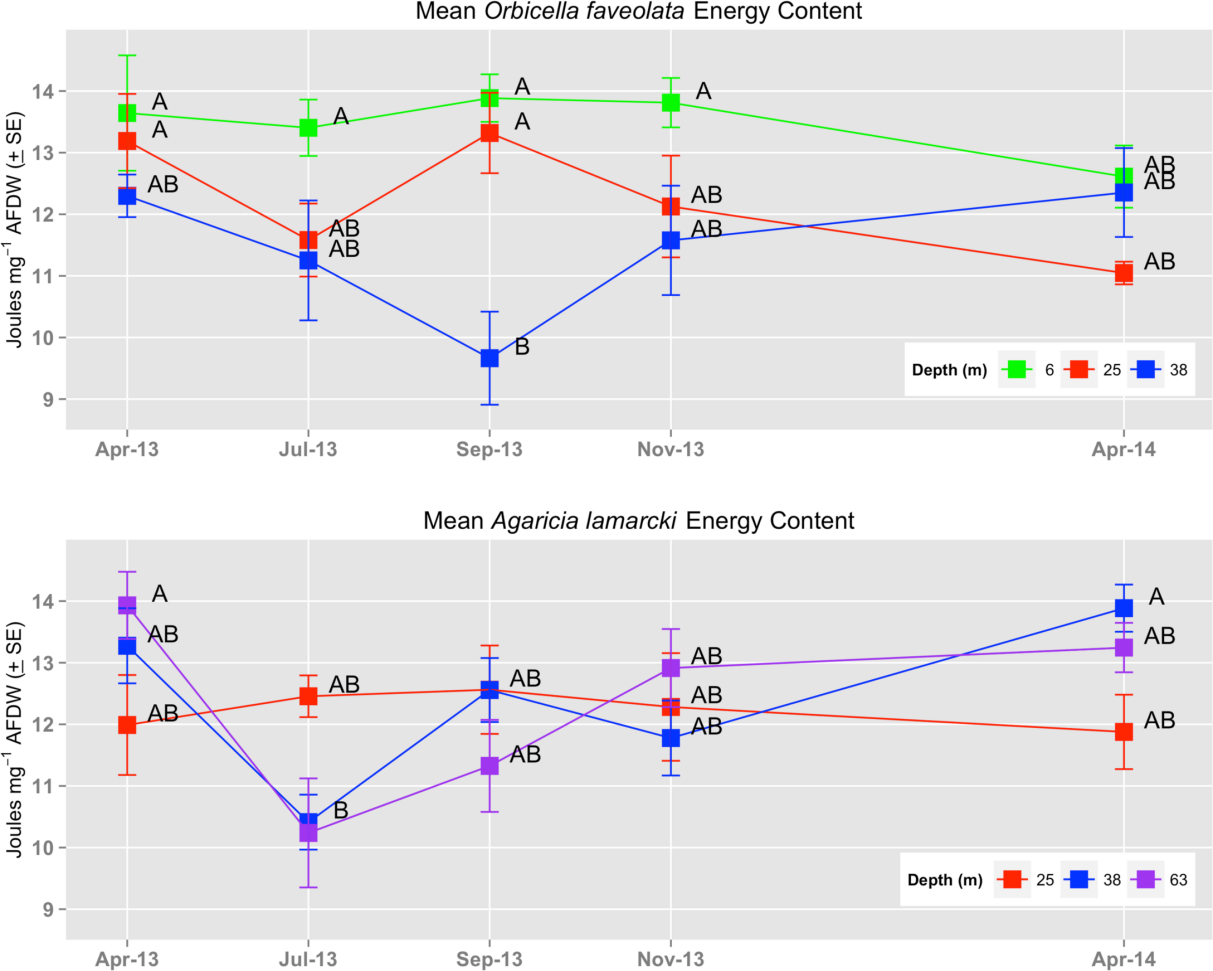
Coral Energy Content. Mean energetic content of *Orbicella faveolata* and *Agaricia lamarcki* subsamples between April 2013 and 2014 from three site and depth levels. Letters adjacent to means values indicate results of a Tukeys HSD post-hoc analysis of the overall interaction between site and sampling period.

The energetic content of *O*. *faveolata* showed stability over time at 6m, varying by only 10.1% (Fig 2). In contrast, both the mid-depth and mesophotic sites exhibited considerable variability, 25m colonies varied by 20.5% and 38m colonies by 27.8% throughout the sampling period. Two-way ANOVA analysis resulted in a significant interaction between Site and Sampling Period (p = 0.032) (Table 2). Tukey’s HSD post-hoc analysis of the interaction indicated that the September 38m data point was significantly lower than a number of other data points, including four of five shallow sampling periods (Fig 2). The energy density of individual *O*. *faveolata* colonies varied two-fold, from a minimum of 7.995 J mg^-1^ ash-free dry weight (AFDW) at the 38m site to a high of 15.859 J mg^-1^ AFDW at the shallowest 6m site—with an overall mean of 12.402 + 0.205 J mg^-1^ AFDW (+ SE).

**Table 2:** Statistical Analyses.

Species	Site p (F_df_)	Period p (F_df_)	Site*Period p (F_df_)
*Orbicella faveolata*	0.00003 (12.43_2,59_)	0.287 (1.28_4,59_)	0.032 (2.30_8,59_)
*Agaricia lamarcki*	0.722 (0.33_2,65_)	0.003 (4.38_4,65_)	0.014 (2.65_8,65_)

Results of Two-Way ANOVA analyses comparing the energy content of coral tissue in *Orbicella faveolata* and *Agaricia lamarcki* across sites and sampling periods.

Shallow colonies of *A*. *lamarcki* (25m) exhibited greater stability through time relative to deeper samples, varying by only 5.8%. Conversely, the 38m and 63m sites varied over the sampling periods by 33.3% and 36.1%, respectively, and had a very similar pattern over time. Two-way ANOVA analysis indicated a significant interaction between Sampling Period and Site (p = 0.014) (Table 2). Tukey’s HSD post-hoc analysis of the interaction effect indicated that energetic content of *A*. *lamarcki* at both mesophotic sites in July 2013 were significantly less than the 63m site in April, 2013 and the 38m site in April 2014. The energy density of individual *A*. *lamarcki* colonies varied two-fold, from a minimum of 8.035 J mg^-1^ AFDW to a maximum of 15.514 J mg^-1^ AFDW, with both extremes occurring at the 63m site. Mean energetic content was 12.346 + 0.189 J mg^-1^ AFDW (+ SE).

### Environmental Characterization

**Fig 3:**
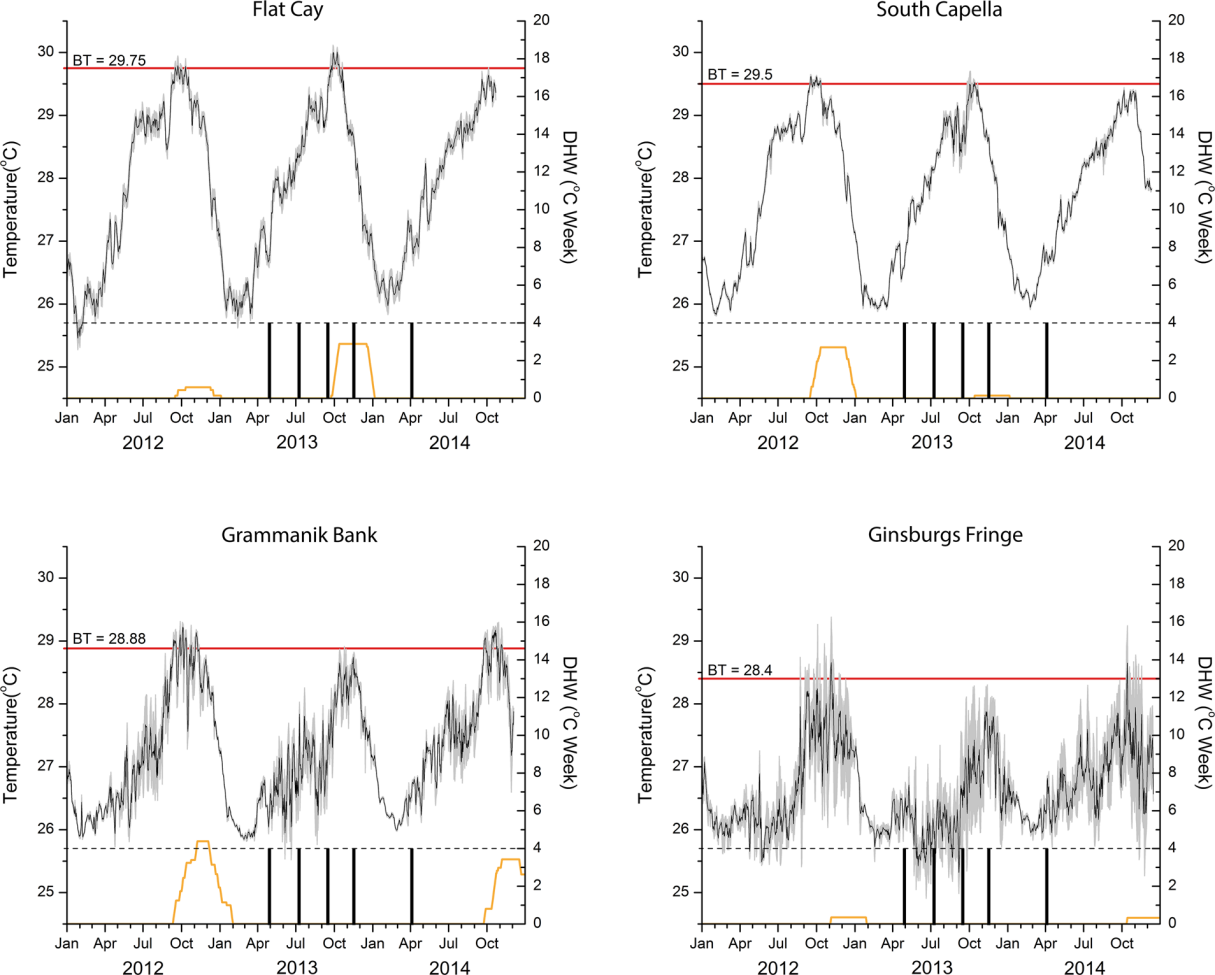
Annual Temperature Records. Mean daily temperature and diel standard deviation (gray shading around mean line). Red line indicates bleaching threshold (BT) as calculated for each site and the BT value (°C) indicated. Yellow lines are calculated degree heating week (DHW) accumulation. Hatched black line indicates the 4 DHW level, suggested as the thermal stress level where bleaching is initiated in coral communities. Vertical black lines denote sampling periods.

Temperature trends varied significantly with time and site (p<0.001; Table 3; Fig 3). Flat Cay (6m) and South Capella (25m) exhibited similar temporal trends for both 2013 and 2014. South Capella, however, experienced reduced thermal peaks when compared to Flat Cay, between the third and fourth sampling events. In 2012, prior to coral colony sampling, South Capella accumulated about 3 DHW of thermal stress, whereas Flat Cay showed almost no thermal stress. The pattern was reversed in 2013, when Flat Cay accumulated about 3 DHW during project sampling and South Capella experienced almost no thermal stress. During this period, temperatures at Flat Cay peaked to roughly 0.5°C higher than at South Capella. However, bleaching at Flat Cay in October was mild (9.8% prevalence) and not very different from other non-bleaching years (10.6% prevalence; mean of years 2009, 2011, and 2012 during the thermal maximum) [34].

**Table 3:** RM-ANOVA Results for Benthic Temperature.

		**Df**	**Sum Sq**	**Mean Sq**	**F-value**	**p-value**
**Date**	Site	3	79	26.376	8.934	<0.0001
	Residuals	1076	3177	2.952		
		**Df**	**Sum Sq**	**Mean Sq**	**F-value**	**p-value**
**Date:Site**	Site	3	955.0	318.3	1166	<0.0001
	Residuals	3021	824.7	0.3		

Repeated measures ANOVA output comparing benthic temperature records at the sampling sites

The mesophotic sites at Grammanik Bank (38m) and Ginsburgs Fringe (63m) showed greater diel temperature variability than their shallow counterparts as well as reduced temperatures throughout both sampling years. In the year prior to sampling (2012), there was over 4 DHW of thermal stress recorded at Grammanik Bank, and this resulted in moderate bleaching (34.8% prevalence) compared to other non-bleaching years (12.0% prevalence, mean of years 2009, 2010, and 2011 during the thermal maximum) [34, 39]. Over the period of coral sampling in 2013 and 2014 there was little or no thermal stress recorded at Grammanik Bank or Ginsburgs Fringe.

**Fig 4:**
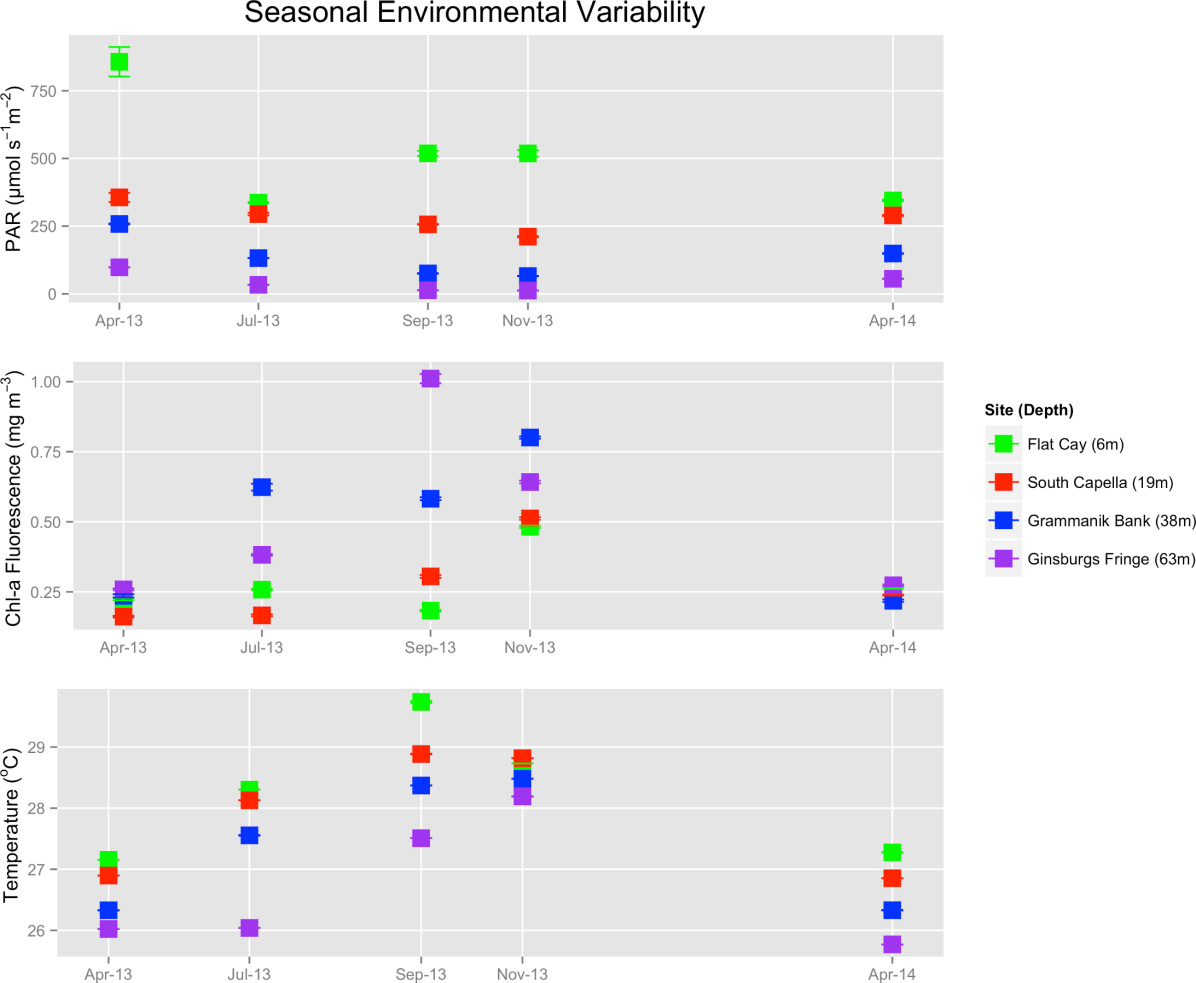
Environmental Characterization of Sampling Sites. PAR, chl-a fluorescence and temperature at sampling locations. Data points indicate the mean value taken within one meter of the sampling depth indicated in the legend.

**Fig 5:**
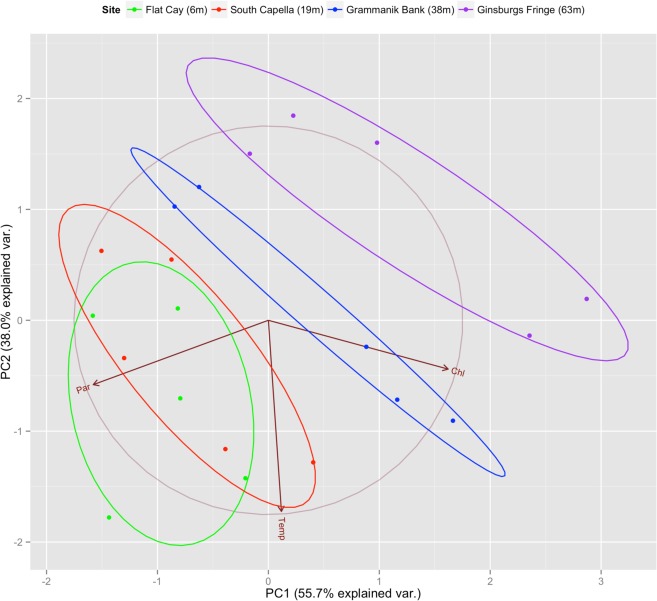
Principle Component Analysis of CTD Data. The first two principle components are indicated here along with arrows showing the influence of PAR, temperature and chl-a. Ellipsoids represent the default 68% confidence interval for each site.

Measurements of PAR, chl-a fluorescence and temperature exhibited considerable variability across the sampling period (Fig 4). Principal component analysis of CTD data indicates a separation between mesophotic and shallow reefs (Fig 5). The first two principle components explain 93.7% of environmental variability at the sampling sites—with MCE sites exhibiting reduced PAR and increased chl-a fluorescence along PC1 and reduced PAR and temperature along PC2 (Fig 5; Table 4). PAR values exhibited statistically significant differences between sites while chl-a and temperature did not (Table 5). Tukey HSD post-hoc comparisons of PAR are found in Table 6 and highlight the light differences between mesophotic and shallow environments. While temperature and chl a were not statistically different between sites, each site exhibited high variability in these variables, with peaks at different periods over the sampling year.

**Table 4:** Principle Components Analysis Results.

**Principle Components**	**PC1**	**PC2**	**PC3**
PAR	-0.697	-0.310	0.647
Chlorophyll-a	0.715	-0.235	0.658
Temperature	0.052	-0.921	-0.386
**Importance of Components**	**PC1**	**PC2**	**PC3**
Standard deviation	1.293	1.068	0.433
Proportion of variance	0.557	0.380	0.063
Cumulative proportion	0.557	0.937	1.000

Results of principle component analysis comparing PAR, chl-a and temperature across all sampling sites and dates.

**Table 5:** ANOVA Results.

		Df	Sum Sq	Mean Sq	F-value	p-value
**Temperature**	Site	3	6.702	2.234	2.028	0.151
	Residuals	16	17.626	1.102		
**Chlorophyll-a**	Site	3	0.250	0.083	1.664	0.215
	Residuals	16	0.802	0.050		
**PAR**	Site	3	636521	212174	15.58	5.26x10^-5^
	Residuals	16	217941	13621		

ANOVA results comparing environmental factors across sites and sampling dates.

**Table 6:** Tukey’s HSD post-hoc Analysis of PAR data.

	diff	lwr	upr	p-value
GNS-FLC	-472.85	-684.03	-261.66	<0.0001
GRK-FLC	-379.28	-590.46	-168.10	0.0005
SCP-FLC	-233.61	-444.80	-22.429	0.0276
GRK-GNS	93.565	-117.62	304.75	0.5953
SCP-GNS	239.23	28.049	450.42	0.0238
SCP-GRK	145.67	-65.516	356.85	0.2385

Tukey’s HSD results for PAR data. GNS: Ginsburgs Fringe, GRK: Grammanik Bank, SCP: South Capella, FLC: Flat Cay. Significance is indicated by asterisks.

**Fig 6:**
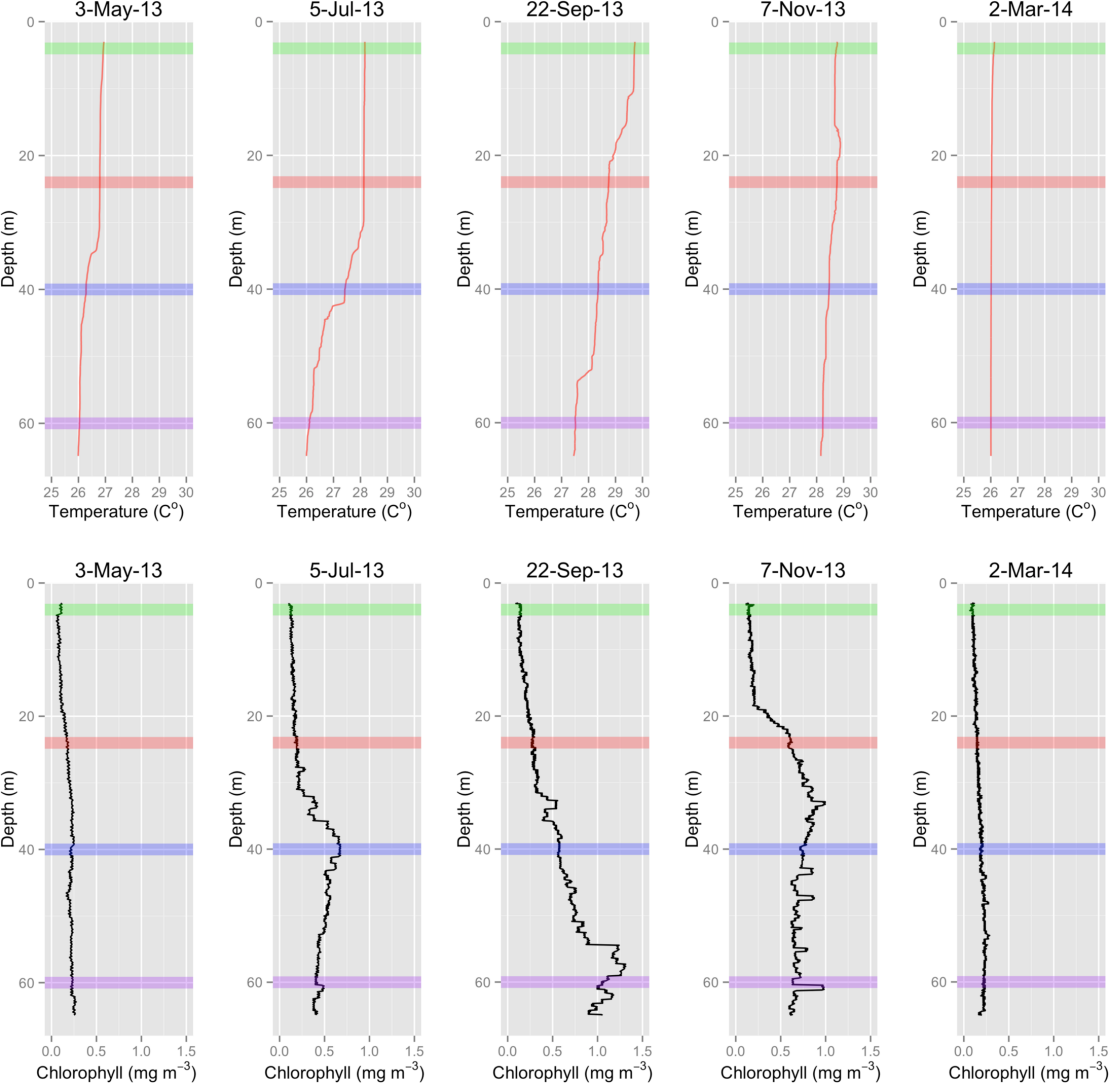
Seasonal Water Column Characterization. Water column temperature and chlorophyll-a fluorescence measurements concurrent with each sampling event. Horizontal colored bars correspond to sampling depths.

Seasonal changes in water column stratification can be identified in vertical profiles taken at the Ginsburgs Fringe site (Fig 6). During the early parts of both 2013 and 2014, the water column was well mixed to 60m depth, indicated by a consistent thermal regime and low variability in chl-a fluorescence. A weak thermocline was evident at 35m in May 2013. As the summer of 2013 progressed, temperatures increased across all depths, but more abruptly shallower than 30m. During July and September thermoclines were present, resulting in a temperature range of 2°C across the sampling depth range. The November 2013 cast showed a return to the well mixed regime measured in both spring samples; however, there were increased temperatures deeper than 30m compared to earlier in the year, most notably at depths exceeding 55m.

**Fig 7:**
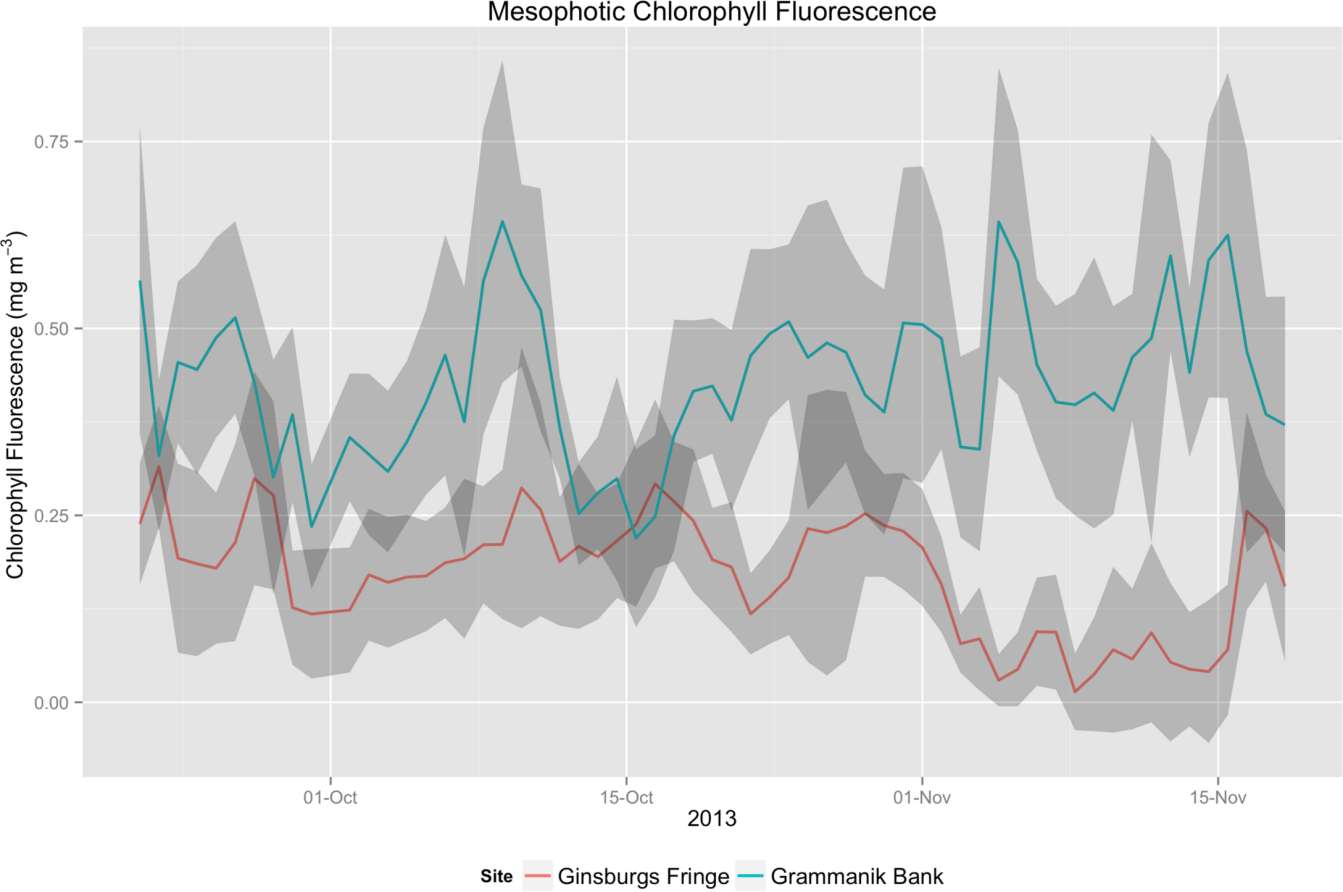
Mesophotic Chlorophyll Fluorescence. Mean daily Chlorophyl-a fluorescence at Grammanik Bank and Ginsburgs Fringe from 9/21/13 to 11/19/13. Shaded regions represent daily standard deviation.

Vertical profiles of chlorophyll-a fluorescence values exhibited similar trends to temperature. The spring casts showed low chl-a fluorescence that were consistent across depths. During July and September, chl-a levels increased with maxima occurring at major thermocline depths. The November cast exhibited relatively consistent and high chl-a levels across the entire depth range below 20m. The chl-a fluorescence maximum in July occurred at the Grammanik Bank sampling site, and the September maximum encompassed Ginsburgs Fringe. Benthic recording of chl-a at these two sites in October and November 2013, between vertical profile sampling, showed that the Grammanik Bank had significantly higher and more variable chl-a values than Ginsburgs Fringe (Fig 7; Mean_Grammanik_ = 0.430 ± 0.180 S.D., Mean_Ginsburgs Fringe_ = 0.171 ± 0.126 S.D; p<0.001 paired t-test). In many cases chlorophyll-a levels at Ginsburgs Fringe were near lower detection limits of the sensors, indicating very low abundance of phytoplankton.

## Discussion

Perhaps the most striking pattern revealed here was increasing seasonal variability of energy content in coral tissues with depth. The shallowest sites for both species exhibited far less change in energetic content through time than deep sites. Furthermore, timing differences between *O*. *faveolata* and *A*. *lamarcki* energy variation across sampling periods, but within the same sites, suggests species-specific factors may be shaping energetic responses of corals inhabiting MCEs. Given the single-year timeframe of this study, we suggest that the main mechanism affecting the measured seasonal energetic drops in mesophotic colonies sampled is likely to be reproduction. It is important to note, however, that depth adaptations in the corals measured likely have a profound impact on the overall energetic measurements made. The refugia potential of mesophotic coral reefs will ultimately rely on the interplay between site-specific environmental characteristics and species-specific symbiont selection and reproductive activities.

### Reproduction

One of the most influential energetic activity corals undertake is that of sexual reproduction. The production of gametes and larvae requires considerable energy investment on the part of the coral. Richmond showed that colonies of *Pocillopora damicornis*, a brooding species in the region he investigated, invest between 2 and 20% of their total energetic content into larvae production during each month of reproduction [40]. In addition, he suggested that *P*. *damicornis* were investing 1–10 times the calories into reproduction as they were into growth. Assuming that the energy demands of reproduction rival those of tissue growth and maintenance, the influence of reproduction on the overall energy content of corals is likely to be important.

The reproductive strategies of *O*. *faveolata* and *A*. *lamarcki* are different and may be very influential in the resilience of both species to future stress events. While reproduction was not measured directly in our study corals, we have inferred reproduction based on annual timing from the literature. *Orbicella* spp. are broadcast spawning species that release egg and sperm bundles that break up and fertilize in the water column [41]. Reproduction in *O*. *faveolata* is expected in either August or September, just prior to the third sampling period in 2013, in both shallow [42] and mesophotic environments [43]. Thus, the drop in energy content exhibited by the 38m colonies in September 2013 coincided with likely gamete release and this may explain the pattern of annual variation.

There are two factors that explain the opposing energetic trends between shallow and mesophotic *O*. *faveolata* during the reproductive period. First, greater solar irradiance allows for higher net productivity in corals at shallow depths [29]. The energy expended in reproductive activities of shallow colonies is likely fully replaced over a short time period by photosynthesis, evinced by the stability of shallow colony energy content throughout the reproductive seasons of both species. Second, it has been shown that gametogenesis in *O*. *faveolata* is delayed in mesophotic colonies relative to shallow colonies, but once initiated is more rapid in mesophotic corals [43]. The authors also showed across our same sampling region on the southeastern Puerto Rican Shelf that mesophotic colonies were hyper-fecund, producing greater numbers of gametes than shallow colonies. It is likely, therefore, that mesophotic *O*. *faveolata* experience a compressed period of strong reproductive activity, incurring the same or greater energy costs as shallow colonies over a much shorter period of time. The September drop in energetic content for mesophotic colonies is likely a result of intense gamete production followed by spawning. The lack of energetic drop in shallow colonies is possibly a result of prolonged and less intense gametogenesis that may be mostly or fully supported by photosynthesis.

At different times throughout the sampling period, *O*. *faveolata* colonies at 25m trended with both shallow and mesophotic colonies. While not statistically significant, the downward trend in energy status of 25m colonies in July, followed by an increase into September is likely due to environmental factors, and not an early spawning event. Spawning of *O*. *faveolata* across its depth range in the Caribbean is timed between August and October [35, 43] and has not been observed earlier, suggesting that early spawning is an unlikely explanation for energy content decreases prior to August. As the well-mixed water column of spring gives way to a stratified regime in summer, it is possible that colonies at South Capella were receiving increased heterotrophic food sources through tidal boring, and then increased sunlight during periods while above the thermocline. Colonies living at 25m appear to experience conditions reminiscent, but not identical, to both shallow and mesophotic reefs at different times of year. More work is necessary to understand the factors affecting colonies residing within the transition zone between shallow and mesophotic reefs.

In contrast to *O*. *faveolata*, *A*. *lamarcki* is a brooding species that undergoes internal fertilization and releases fully competent larvae during planulation [44]. The timing of reproduction in *A*. *lamarcki* is unknown, but it has been suggested that planulation may occur during the spring alongside other deep-living Caribbean agariciids [45–46]. The energetic minimum exhibited by mesophotic colonies in July 2013 supports the assertion that *A*. *lamarcki* are reproducing in the first half of the year. The disparity between shallow and mesophotic energetics during reproduction is likely attributable again to differences in photosynthetic net productivity. Shallow colonies experiencing higher light levels may be capable of supporting reproduction without marked losses of energy while mesophotic colonies are not. In addition, as may be the case with *O*. *faveolata*, mesophotic *A*. *lamarcki* may be placing greater energetic investment into reproduction for unknown reasons.

The timing of reproduction influences the extent to which energy content is affected in both species. Mesophotic *O*. *faveolata* and *A*. *lamarcki* both experienced similar energetic drops during their reproductive periods; however, since *O*. *faveolata* spawns in the fall, colonies may be at greater risk of disturbance in future stress events. Thermal stress across all sampling sites generally begins in the second half of September and continues through January (Fig 3). *O*. *faveolata* experience their energetic minimum in September, at the beginning of the thermal stress season. If algal symbionts are thermally stressed and this leads to a reduction in photosynthetic subsidies, such as occurs during bleaching events, then mesophotic corals may be more susceptible to mortality. Conversely, mesophotic *A*. *lamarcki* have considerably more time for energetic recovery following a July energetic minimum—with colonies exhibiting greater energetic content in September than *O*. *faveolata*.

### Respiration, Photosynthesis, and Trophodynamics

Along with reproduction, variability in the environmental and physiological conditions across a depth gradient likely affects energy content of coral colonies. The effects of environmental variability and depth on coral growth are well reviewed in [47]. As light attenuates, corals adapt—exhibiting reduced calcification and denser skeletons [48–49]. Colonies display flattened, plating growth forms intended to better capture light and compensate for reduced photosynthesis to respiration ratios (P/R ratio) [29, 50]. The stability of energy content in shallow corals in this study suggests that colonies at those depths may be maintaining positive net productivity throughout the year. High light levels and low short-term thermal variability at the shallowest sites may assist in maintaining more constant energy levels.

Conversely, mesophotic colonies of both species are likely to receive less light and experience greater short-term thermal variability. *O*. *faveolata* and *A*. *lamarcki* have been shown to modulate symbiont communities at mesophotic depths—favoring more productive but less thermally tolerant clades when light and temperature are reduced [31, 51–52]. Adapting zooxanthellate communities at depth is likely an effort to increase photosynthetic production in reduced light. While both subject species exhibit differing symbiont communities with depth, it is possible that they possess unequal heterotrophic capabilities. While *A*. *lamarcki* has been shown to successfully feed at mesophotic depths [32], the heterotrophic plasticity of *O*. *faveolata* is currently unclear—although Lesser and others have suggested that it may be less suited to heterotrophy than other coral species [53].

Whether *O*. *faveolata* is feeding heterotrophically or not, the seasonal drop in energy content of mesophotic colonies indicates that the combination of more productive symbionts with increased planktonic food sources in the water column cannot sufficiently support reproductive activities in deep living colonies without a drop in energetic content. Similarly, the precipitous seasonal energetic drop in *A*. *lamarcki*, followed by rapid energy recovery likely suggests that the combination of shifting symbiont communities and increased heterotrophic feeding does not allow mesophotic colonies to maintain consistent energy throughout the year. Ultimately, the timing of the energetic drop in both species—likely linked to reproductive activities—defines the energetic state of these species as they enter the season most associated with thermal stress events.

Another possible explanation for the low energetic variability exhibited by shallow colonies versus mid and deep colonies has to do with historic seasonal dynamics. Specifically, the history of bleaching and coral mortality at 6m is considerably different than at the other three sites. Thermal stress events affected shallow water corals in 2005 and 2010 [34], but the effect of thermal stress declined with depth [39]. Also, while there was mesophotic bleaching in 2012, it was not severe and did not result in loss of coral cover [39]. As such, it is possible that differential mortality has occurred between sites. It may be that the only surviving shallow colonies are those that had the most efficient energy maintenance regimes going into previous stress events, and therefore they showed constant energy content over the sampled year.

### MCEs as Refugia

The seasonal energetic content of *O*. *faveolata* and *A*. *lamarcki* suggest differing refuge potential for each species in MCEs habitats. *O*. *faveolata* appears to be better adapted for shallow water living, and mesophotic colonies—though prevalent—may be at risk of future disturbance. Corals incur large energy costs during reproduction [40] and deep-living colonies appear to require a considerably longer recovery period than shallow colonies. In *O*. *faveolata* reproduction, energy content minima, and the annual thermal maximum period all coincide. Thus, the corals have the lowest energy stores during the period when there could be high temperature caused stress. If during this period there is photosynthetic stress and bleaching that limits energy production, energy stores may be insufficient to maintain colony vitality. In contrast, spring brooding and subsequent energetic minimum exhibited by *A*. *lamarcki* colonies may give them time to recover energy content prior to the annual thermal maximum. As such, *A*. *lamarcki* colonies living at or beyond 40m have a life history that allows them greater energy stores during the most stressful time of year.

The conclusion that MCEs may represent a better refuge for *A*. *lamarcki* than *O*. *faveolata* needs to be balanced by investigations into the ability of mesophotic larvae and newly recruited corals to survive stress events. Studies have shown that increased temperatures at the time of larval release and settlement can impact survivorship in coral larvae and recruits [54–56]. While the timing of *A*. *lamarcki* reproduction provides abundant energy stores for extant colonies entering the thermal maximum, larvae and newly recruited colonies are likely to experience stress shortly after settling. Alternatively, *O*. *faveolata* larvae may have the opportunity to settle shortly after the thermal maximum, providing new colonies a greater period of time to grow and store energy. The individual ways that coral species time life history events in relation to stressful conditions may contribute to the species-specific responses of corals to climate change—and likely affect the refugia potential of MCEs.

## Supporting Information

S1 Supporting Information A comparison of shallow water environments at Flat Cay and Buck Island, St. Thomas, US Virgin Islands. (PDF)
