# Supplementary material for: Seasonal Variability in Calorimetric Energy Content of Two Caribbean Mesophotic Corals
Source: PLoS One. 2016 Apr 6;11(4):e0151953. doi: 10.1371/journal.pone.0151953 (PMC4822962; doi:10.1371/journal.pone.0151953)
Supplement: S1 Supporting Information — (PDF) [file pone.0151953.s001.pdf]

S1 Supporting Information. A comparison of shallow water environments at Flat Cay and Buck Island, St. Thomas, US Virgin Islands

The environmental characteristics at Flat Cay and Buck Island were compared using data from four CTD casts taken between September, 2007 and October, 2011. Temperature and chlorophyll-a fluorescence were compared at 5m depth for both locations using paired t-tests. No significant difference was found, justifying the combination of energetic measurements at both sites.

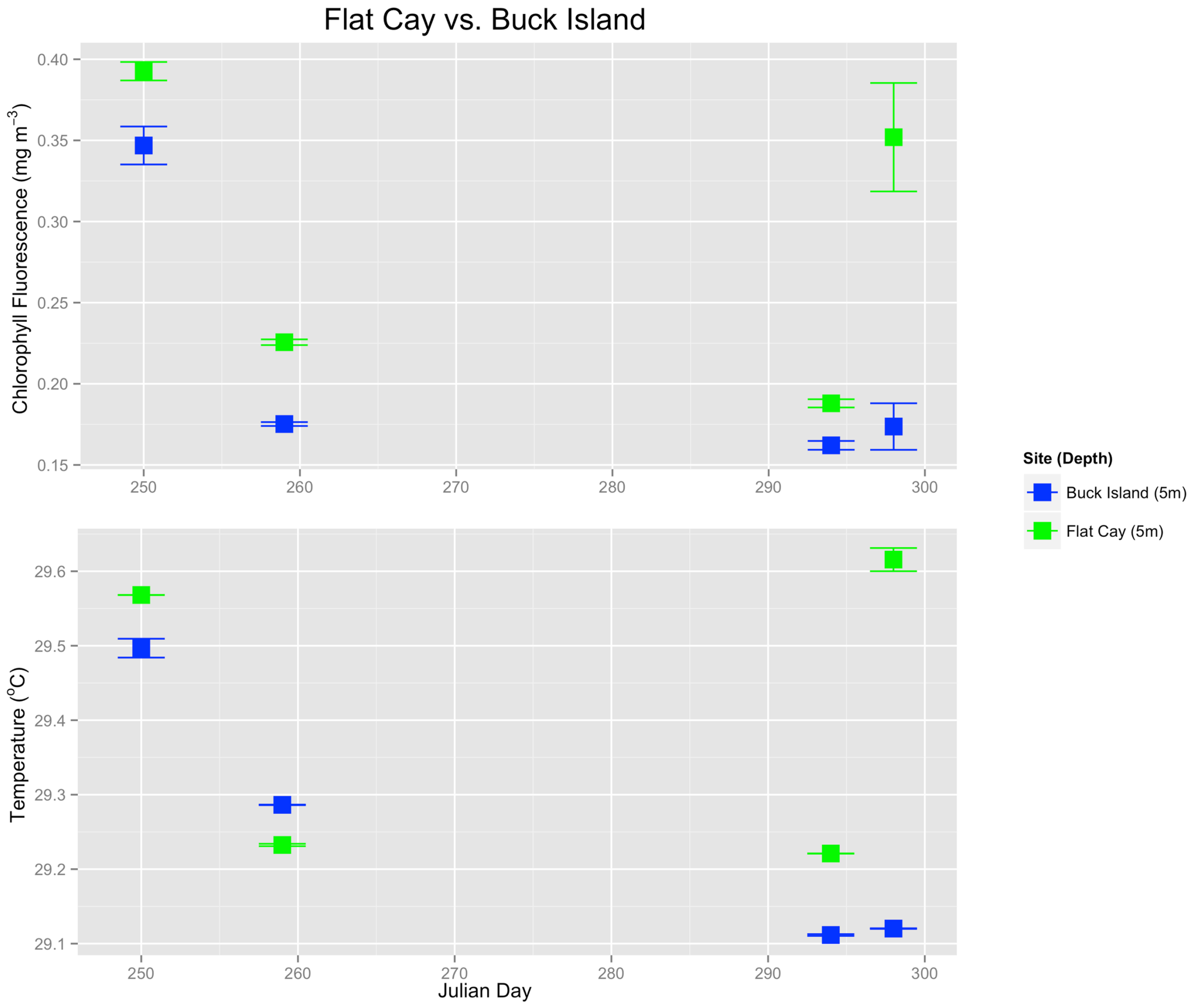

Figure A. Chlorophyll-a Fluorescence and Temperature at Buck Island and Flat Cay
Mean temperature and chl-a fluorescence in four concurrent CTD casts at Buck Island and Flat Cay, St. Thomas. Buck Island is colored in blue, Flat Cay in green. Error bars indicate standard error.

Table A. Statistical Analyses of Chlorophyll-a fluorescence and Temperature

Chlorophyll-a Fluorescence		
t-value	df	p-value
2.157	3	0.1199

Temperature		
t-value	df	p-value
1.313	3	0.2806

Paired t-test results for Chl-a fluorescence and temperature at Buck Island and Flat Cay.
